# Response to the Letter to the Editor Entitled “Interleukin-18: A Critical Culprit in Inflammatory Renal Disease”

**DOI:** 10.1016/j.ekir.2026.106341

**Published:** 2026-02-17

**Authors:** Elena-Bianca Barbir, Anukul Ghimire, Taryn Stokowski, Pram Thennakoonwela, Rebecca Brassington, Wirongrong Churngchow, Samih H. Nasr, Manjula Gowrishankar, Ainslie Hildebrand, Kim Solez

**Affiliations:** 1 Department of Laboratory Medicine and Pathology, University of Alberta, Edmonton, Alberta, Canada; 2 Department of Medicine, University of Toronto, Toronto, Ontario, Canada; 3 Anatomic Pathology, Mayo Clinic, Rochester, Minnesota, USA

The author replies:

We read with great interest the letter to the editor entitled “Interleukin-18: A critical culprit in Inflammatory Renal Disease,”^1^ which is a comment on our case report entitled “IgA-Dominant Mesangial Proliferative Glomerulonephritis in Prolidase Deficiency.”^2^ In the letter, the author suggested ordering interleukin (IL)-18 in our patient with prolidase deficiency and IgA-dominant mesangial peripheral glomerulonephritis, and described the pathophysiology of IL-18 in numerous diseases associated with inflammation.

We agree with Dr. Sergi that for select patients, there is an emerging role for serial cytokine monitoring in multiple immune-mediated diseases, because these represent a potential noninvasive strategy to assess response to therapy, and in certain instances, it may help with therapy selection.^3^ Barriers to implementation in the Canadian context include the following: (i) the absence of routine access to cytokine panels or single cytokine testing, though several send out options do exist often with prohibitive costs associated, and (ii) the absence of clinical validation of these tests, rendering interpretation challenging. This highlights the need for more research to allow us to move toward future clinical implementation. The data Dr. Sergi references for patients with IgA nephropathy certainly demonstrates an association between serum IL-18 levels and clinical disease severity, and warrants the pursuit of larger studies. Because prolidase deficiency is a rare disease, data from other validation cohorts (IgAN, potentially systemic lupus erythematosus) may need to be extrapolated for patients with prolidase deficiency, though the case report he referenced, by Alrumayyan *et al.*^4^ does identify the potential for serum IL-18 levels to identify a component of autoimmunity in PD cases.

The authors described IL-18 as a rather nonspecific inflammatory marker that can be elevated in numerous conditions or states of inflammation. As described in the article, our patient had a complicated hospital stay and thus had many reasons for inflammation. How exactly an IL-18 level would have aided in evaluation of her kidney disease is unclear.

Therefore, though we appreciate the potential utility of an IL-18 level in assessing states of inflammation, its clinical applicability at present is limited. Until our understanding in this area evolves further, ordering this test would only increase health care costs without adding much value.

## Disclosure

All the authors declared no competing interests.

## References

[1] Sergi C.M. (2026). Interleukin-18: A critical culprit in inflammatory renal disease. Kidney Int Rep.

[2] Stokowski T., Thennakoonwela P., Brassington R. (2025). IgA-dominant mesangial proliferative glomerulonephritis in prolidase deficiency. Kidney Int Rep.

[3] Barbir E.B., Mohan A., Koirala P. (2025). Biomarker-guided infliximab therapy for immune checkpoint inhibitor-induced acute interstitial nephritis. Nephrol Dial Transplant.

[4] Alrumayyan N., Slauenwhite D., McAlpine S.M. (2022). Prolidase deficiency, a rare inborn error of immunity, clinical phenotypes, immunological features, and proposed treatments in twins. Allergy Asthma Clin Immunol.

